# Ketamine-assisted buprenorphine initiation: a pilot case series

**DOI:** 10.1186/s13722-024-00494-2

**Published:** 2024-08-29

**Authors:** Lucinda A. Grande, Tom Hutch, Keira Jack, Wendy Mironov, Jessica Iwuoha, Martin Muy-Rivera, Jacob Grillo, Stephen A. Martin, Andrew Herring

**Affiliations:** 1 Pioneer Family Practice, 5130 Corporate Ctr Ct SE, Lacey, Washington 98503 USA; 2 We Care Daily Clinics, Auburn, Washington USA; 3 Conquer Addiction PLLC, Monroe, Washington USA; 4 UMass Chan Medical School, Worcester, USA; 5 Alameda Health System, Oakland, California USA; 6 Department of Family Medicine, University of Washington School of Medicine, 1959 N.E. Pacific St., Box 356390, Seattle, WA 98195-6390 USA

**Keywords:** Buprenorphine initiation, Ketamine, Fentanyl, Methadone, Precipitated withdrawal

## Abstract

**Background:**

Many people with opioid use disorder who stand to benefit from buprenorphine treatment are unwilling to initiate it due to experience with or fear of both spontaneous and buprenorphine-precipitated opioid withdrawal (BPOW). An effective means of minimizing withdrawal symptoms would reduce patient apprehensiveness, lowering the barrier to buprenorphine initiation. Ketamine, approved by the FDA as a dissociative anesthetic, completely resolved BPOW in case reports when infused at a sub-anesthetic dose range in which dissociative symptoms are common. However, most patients attempt buprenorphine initiation in the outpatient setting where altered mental status is undesirable. We explored the potential of short-term use of ketamine, self-administered sublingually at a lower, sub-dissociative dose to assist ambulatory patients undergoing transition to buprenorphine from fentanyl and methadone.

**Methods:**

Patients prescribed ketamine were either (1) seeking transition to buprenorphine from illicit fentanyl and highly apprehensive of BPOW or (2) undergoing transition to buprenorphine from illicit fentanyl or methadone and experiencing BPOW. We prescribed 4–8 doses of sublingual ketamine 16 mg (each dose bioequivalent to 3–6% of an anesthetic dose), monitored patients daily or near-daily, and adjusted buprenorphine and ketamine dosing based on patient response and prescriber experience.

**Results:**

Over a period of 14 months, 37 patients were prescribed ketamine. Buprenorphine initiation was completed by 16 patients, representing 43% of the 37 patients prescribed ketamine, and 67% of the 24 who reported trying it. Of the last 12 patients who completed buprenorphine initiation, 11 (92%) achieved 30-day retention in treatment. Most of the patients who tried ketamine reported reduction or elimination of spontaneous opioid withdrawal symptoms. Some patients reported avoidance of severe BPOW when used prophylactically or as treatment of established BPOW. We developed a ketamine protocol that allowed four of the last patients to complete buprenorphine initiation over four days reporting only mild withdrawal symptoms. Two patients described cognitive changes from ketamine at a dose that exceeded the effective dose range for the other patients.

**Conclusions:**

Ketamine at a sub-dissociative dose allowed completion of buprenorphine initiation in the outpatient setting in the majority of patients who reported trying it. Further research is warranted to confirm these results and develop reliable protocols for a range of treatment settings.

## Introduction

Buprenorphine reduces risk of opioid overdose death by 50% or more for people with opioid use disorder. [1]. Its use is less encumbered by the strict regulatory constraints that limit access to methadone, the other medication with similar mortality reduction. Yet many people with opioid use disorder who stand to benefit from buprenorphine treatment do not attempt to initiate it due to experience with or fear of spontaneous opioid withdrawal or buprenorphine-precipitated opioid withdrawal (BPOW) [2, 3].

Buprenorphine, a mixed agonist-antagonist at the mu-opioid receptor (mOR) can precipitate distressing withdrawal symptoms when a mOR agonist agent such as morphine, fentanyl, or methadone already occupies the receptors [4]. Avoiding acute-onset withdrawal symptoms after a buprenorphine dose usually requires a preceding period of opioid abstinence. The necessary duration of abstinence, as well as the severity and duration of withdrawal symptoms, depend in part on the buprenorphine dose and varies widely between individuals and between mOR agonist agents. [4]

People who have been using illicit fentanyl and its analogues (hereafter referred to as “fentanyl”) can experience intolerable symptoms from both spontaneous opioid withdrawal and BPOW [2, 3]. Both spontaneous withdrawal and vulnerability to severe BPOW can last for 7 days or longer due to fentanyl’s high mOR affinity and intrinsic efficacy [4], along with its high lipophilicity with associated prolonged elimination [4, 5]. Transition to buprenorphine from methadone also involves a prolonged period of withdrawal risk due to methadone’s long elimination half-life [6].

A variety of strategies have emerged during the fentanyl era to reduce the severity or duration of discomfort during buprenorphine initiation [7], for example (1) low-dose buprenorphine with opioid continuation (also known as “microdosing”), (2) rapid high-dose buprenorphine initiation after opioid discontinuation (also known as “macrodosing”), (3) injection of extended-release buprenorphine after opioid discontinuation [8], and (4) high dose buprenorphine rescue after naloxone self-administration [9]. The first strategy prolongs the duration of the buprenorphine initiation without ensuring success. The latter three strategies ensure a rapid buprenorphine initiation but may incur moderate to severe BPOW at least briefly, with mild discomfort perhaps lasting days. Adjunctive medications such as baclofen, benzodiazepines, clonidine, dicyclomine, gabapentin, hydroxyzine, ibuprofen, lofexidine, loperamide, ondansetron, pramipexole, and trazodone [7, Appendix D] can help relieve symptoms in the outpatient setting but are inadequate for many individuals. The real-world effectiveness of these initiation strategies in the outpatient setting is uncertain [7].

Ketamine is a Schedule III controlled substance with FDA approval for intravenous use as a dissociative anesthetic [10, 11] and for procedural sedation [12] and has a variety of off-label uses. For example, at a dissociative sub-anesthetic dose, it provides rapid relief from treatment-resistant depression [13] and alleviates both acute and chronic pain [14]. Early pre-clinical and clinical research has shown potential for the treatment of anxiety [15], post-traumatic stress disorder [16], and substance use disorder [17].

Although the ketamine molecule interacts with many receptor types, its primary pharmacological mechanism of action is thought to be its antagonist effect at the NMDA-type glutamate receptor (NMDAr) [18]. Blocking the NMDAr rapidly reverses central nervous system adaptations mediating opioid dependence and tolerance [19]. In addition, ketamine rapidly increases both the concentration and efficacy of endogenous opioids, thereby potentially functioning as a proxy opioid agonist [20–22].

An anesthesiology team first explored therapeutic use of ketamine in opioid-dependent patients in a randomized placebo-controlled trial with 50 patients undergoing withdrawal management under anesthesia [23]. Compared to the placebo group, the group that received an anesthetic-dose ketamine bolus and continuous infusion showed significantly reduced signs of opioid withdrawal, including lower blood pressure, heart rate, and serum cortisol levels.

Buprenorphine prescribers may benefit from adding ketamine to their initiation toolkit. In clinical case reports of opioid-dependent patients experiencing intractable BPOW, an intravenous infusion of a sub-anesthetic dose of ketamine immediately terminated BPOW in a pain clinic [24], an emergency department [25], and a hospital inpatient ward [26].

However, in the outpatient setting, where most patients undergo buprenorphine initiation, access to intravenous infusion is not feasible. Additionally, the dose range of those case reports involves an altered dream-like state that would leave an unprotected patient vulnerable to harm. An effective and safe strategy for the use of ketamine to limit or prevent withdrawal symptoms in the outpatient setting could reduce patient apprehensiveness, lowering the barrier to buprenorphine initiation.

Ketamine is commercially available only as an injectable liquid, but can be prepared by prescription at compounding pharmacies as sublingual troches (lozenges), syrup, or other formulations [18]. It can be prescribed off-label for outpatient use by any DEA-licensed prescriber. It does not cause respiratory depression except at doses well above that required for anesthesia [10].

A sub-dissociative dose of oral ketamine, self-administered daily, can relieve chronic pain without noticeable cognitive or perceptual effects, as described in a review of 22 case reports and case series [27]. Among the 166 cases in that review, the effective oral ketamine dose ranged from 15 to 125 mg, bioequivalent to 0.05–0.38 mg/kg, self-administered up to six times daily. Following this model, one of the authors (LG), a family physician and addiction specialist with anesthesiology training, began developing experience in the primary care setting in 2012, aiming for a sub-dissociative dose range. She ultimately prescribed oral or sublingual ketamine to over 600 patients for pain and/or depression over 12 years, and found an effective dose range of 4 to 128 mg, self-administered up to six times daily, similar to the published reports. Upon learning of the success of ketamine infusion for treating BPOW in the emergency department [25], she began exploring the potential of sub-dissociative dose ketamine for buprenorphine initiation in the outpatient setting.

After an initially promising result with LG’s first patient, the Medical Director (TH) of a nearby Opioid Treatment Program (OTP) began collaborating with her by arranging for referral of suitable patients. Here we describe the results of ketamine treatment in a series of patients undergoing buprenorphine initiation.

## Methods

### Terminology

Both buprenorphine and buprenorphine-naloxone are referred to as buprenorphine, with specific formulations listed in Table 2.

### Patients and prescribers

Patients selected for ketamine prescription were either (1) seeking transition to buprenorphine from fentanyl and highly apprehensive of withdrawal symptoms after previous incomplete attempts, or (2) undergoing transition to buprenorphine from fentanyl or methadone and experiencing severe withdrawal symptoms. At the time of data analysis, LG conducted a registry search of the clinic’s electronic health record to identify patients prescribed ketamine between 5/24/2022 and 7/28/2023 for assistance with buprenorphine initiation. She entered these patients, along with patients #2 and #4 (prescribed ketamine by KJ and JG, respectively) into an Excel spreadsheet. She reviewed clinic notes, prescription records, email exchanges between herself and the other prescribers, and text message exchanges with patients to complete the spreadsheet. The co-authors provided clarification and missing details on their patients.

Most patients were prescribed buprenorphine by WM, KJ, JI or MMR at We Care Daily Clinics (WCDC), an accredited and federally certified OTP, and then were referred to LG for ketamine prescription. Patient #1 was prescribed buprenorphine at another clinic and was referred to LG by the patient’s mother for assistance managing withdrawal. Some later patients were referred to LG for both buprenorphine and ketamine by local outreach workers or by earlier patients in the treatment series. Two patients were prescribed both buprenorphine and ketamine by another prescriber under LG’s guidance: patient #2 by KJ at WCDC and patient #4 by JG, sole proprietor of Conquer Addiction PLLC in Monroe, WA, an office-based opioid treatment (OBOT) clinic. Adjunctive medications were prescribed by the buprenorphine prescriber, LG, or both. LG was the sole prescriber of clonazepam. Medicaid covered the cost of buprenorphine and adjunctive medications for all patients.

### Ketamine dose selection

From clinical experience, LG found that the oral ketamine dose at which dissociative cognitive effects began to occur varied between individuals; these effects could be avoided completely by slow upward titration and spacing doses by at least one hour. She identified patient characteristics associated with benefit from a higher starting dose while avoiding cognitive effects, and developed a dosing protocol based on points assigned for the following factors: high opioid tolerance, good general health, severe depression or pain, age less than 65, low anxiety, and no history of drug sensitivities. Weight did not seem relevant except in cases of BMI 40 or higher. For the current treatment trial, the selected starting dose was 16 mg for highly opioid tolerant patients in good general health with severe depression or pain and without limiting factors., This dose could likely be administered up to four times daily without dissociative effects, with suitable spacing between doses. In a 70 kg person, with a bioavailability of approximately 25% [18], ketamine at 16 mg administered sublingually is equivalent to less than 0.06 mg/kg administered intravenously, representing 3–6% of an anesthetic dose [10] and 14% of the standard infusion dose (0.5 mg/kg) for treatment-resistant depression [13].

### Ketamine number of doses

LG initially prescribed ketamine to be dispensed as four 16-mg doses. However, she found that more doses were usually required to complete initiation, and access to a refill was almost always limited by a lack of transportation and/or incompatible pharmacy hours. She therefore increased the prescription to eight doses in most cases (except only four for patient #20, reason undocumented). She set a limit of eight doses, as using all at once instead of as directed would result in a total of 128 mg, which is less than the typical amount needed for one recreational experience [28] (see Box with ketamine bioequivalent doses for different uses). LG weighed the risk of misuse causing a single episode of temporary incapacitation (considered low in these highly motivated patients) against the daily risk of overdose death from continued fentanyl use.


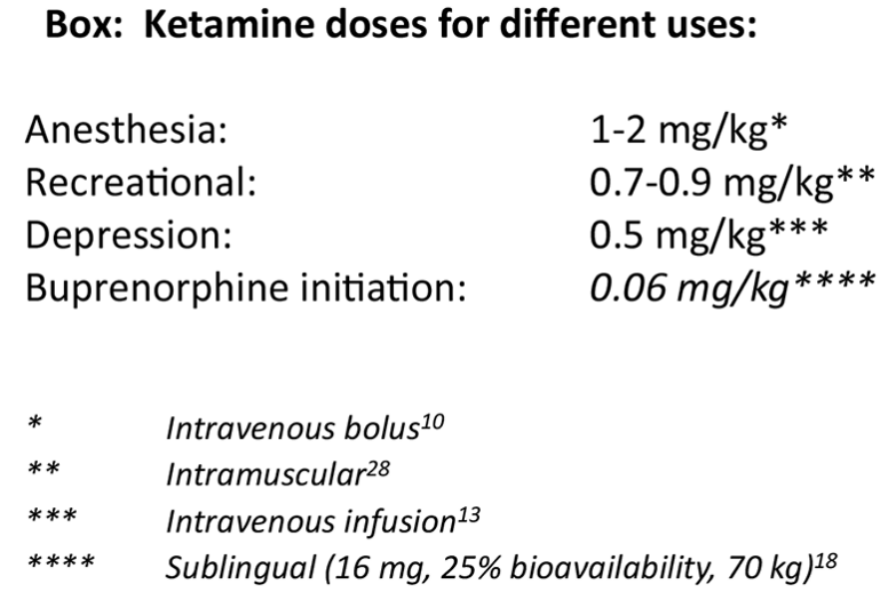


### Monitoring

Patients self-administered all medications at home. LG managed the transition process mostly via telehealth (HIPAA-compliant video calls, telephone, or text messaging on a near-daily, daily, or multiple-times-per-day basis), with occasional in-person visits. Additionally, WCDC patients visited the OTP sometimes repeatedly during the transition process. LG and the WCDC team maintained close communication. Baseline urine drug testing was performed at WCDC per opioid treatment program regulations. Follow-up urine drug testing was not conducted as it was not part of routine care and would not have altered treatment plans.

### Compounding

Quality Compounding Solutions in Kent, WA prepared a batch of 16-mg ketamine troches to be dispensed in packets of four for $12.50 or eight for $15. Some patients were dispensed ketamine from other compounding pharmacies as 16 mg/ml syrup to be self-administered sublingually with a 1 ml dropper (with unit markings to allow 0.5 ml dosing), at a price of $20–25 for 8 ml. A typical prescription was written as follows: ketamine 16 mg troche [or 16 mg/ml syrup] 8–16 mg sublingual 2–3 times per day as needed for withdrawal symptoms, maximum 48 mg/day, quantity 8 troches [or 8 ml].

Since insurance typically does not cover compounded medications, WCDC or LG absorbed the cost of the ketamine, integrating it into their existing budgets without additional cost to patients. To overcome patient transportation barriers, WCDC transported some patients to the pharmacy in a van. Postal mail was avoided due to delay and unreliable postal addresses.

### Initial inclusion criteria

Patients were considered for ketamine treatment if they met the following criteria: (1) requesting transition to buprenorphine from fentanyl, (2) age 60 or younger, (3) no active cardiac disease or uncontrolled bipolar or psychotic disorder, and (4) a reliable shelter and phone number. Exceptions were made for patients #17, #29, #31 and #34 (transition to buprenorphine from methadone), patient #21 (age 62), and patients #18 and #29 (cardiac dysrhythmia discovered while on methadone treatment) after consideration of relative risks of treatment vs. non-treatment for those patients.

### Informed consent

During the initial consultation, LG explained that use of ketamine was off-label for this indication and exploratory in nature, the effective dose and dose timing was unknown, and side effects such as dissociation could occur but were unlikely at the prescribed dose. Patients referred from WCDC were additionally provided a written explanatory handout. Patients could ask questions, and patient preferences regarding the pace of initiation and adjunctive medications were usually accommodated. The patients signed a release of information form at the time of referral from WCDC to LG, and these were retained in the patient’s medical record at both sites. We did not ask patients to sign informed consent as they were not enrolled in formal research.

### Patient instructions

The treatment strategy evolved over the course of 14 months, as described in the Discussion. Individual patients’ treatment response led to adjustments during their treatment. Many of the earlier patients were instructed to use ketamine as a supplement to a low-dose initiation protocol previously established at WCDC, which involved a steady increase in buprenorphine dose over 10 days while supplementing with methadone dispensed daily at the OTP, or with continued illicit fentanyl use, or both. Patients were also encouraged to use adjunctive medications (clonidine, gabapentin, etc.) if needed. We offered and encouraged extended-release buprenorphine (XR-BUP) following completion. LG asked patients to make notes on doses, timing, adjunctive medications and symptoms and to remain in contact daily. Patients recorded these notes inconsistently, so only major features of treatment in each case are available and shown in Table 2.

### Initiation outcome categories

Outcome categories were established at the time of data analysis to describe meaningful distinctions in treatment outcomes. Patients who “completed initiation” were those who reported use of ketamine and were later able to tolerate buprenorphine ≥ 8 mg within a 24-hour period without worsening of withdrawal symptoms. The remaining patients were divided into two groups: (1) those who reported trying ketamine but did not complete initiation, and (2) those with “no outcome information.” Patients in the second group were either (1) not dispensed ketamine per the state Prescription Monitoring Program (PMP) or (2) dispensed ketamine per the PMP and either (a) denied using it or (b) could not be contacted after attempts on at least two days. Patients with 30-day retention status were those who remained in contact with the team on or after 30 days after completing initiation, and reported continued use of buprenorphine at that time.

### “Earlier” and “Later” categories

At the time of data analysis, the 24 patients who confirmed using ketamine were divided into an “Earlier” group (8 patients starting before 2/23/2023) and a “Later” group (16 patients starting on or after 2/23/2023). The chosen boundary was the starting date of the first patient who discontinued fentanyl and used ketamine to manage withdrawal symptoms, a strategy which later became a routine component of initiation instructions because of its observed effectiveness.

### Ethical considerations

Institutional Review Board exemption was obtained from WCG IRB (Puyallup, WA) for publication of de-identified case descriptions.

## Results

Self-reported patient demographic and clinical characteristics of all patients who received a ketamine prescription are provided in Table 1, key treatment elements and outcomes for each patient for whom outcome information was available is in Table 2, and a summary of results in Table 3.

**Table 1:** Self-reported patient demographic and clinical characteristics*

ID	Age	Sex	Referral source	Rx date	Prev bupe (years)	Opioid years	Opioid injection	Fentanyl (months)	Daily fentanyl	Highest MTD dose
1	26	F	OBOT*	5/24/2022	0	1.3	no	6	10 pills	0
2	38	M	OTP	6/7/2022	1	22	yes	unknown	3–5 pills	75
3	37	F	OTP	6/8/2022	PW	6	no	24	6–8 pills	unk
4	25	F	OBOT	6/12/2022	0.5	1.5	no	18	20 pills	0
5	19	F	OTP	12/5/2022	0	0.5	no	4	2–8 pills	0
6	37	M	OTP	12/6/2022	0	22	yes	12	20 pills	50
7	28	F	OTP	12/16/2022	0	11	yes	12	10–20 pills	unk
8	42	F	OTP	12/22/2022	0	1	no	2	4–6 pills	60
9	35	F	OTP	1/19/2023	5	14	no	12	20 pills	215
10	39	F	OTP	1/30/2023	intermittent	13	yes	24	5 pills	0
11	43	F	OTP	1/31/2023	0.5	18	no	13	4–5 pills	130
12	41	F	OTP	2/3/2023	3	20	yes	18	1–2 points	0
13	27	F	OTP	2/23/2023	0	1.25	no	15	10 pills	30
14	45	F	OTP	3/3/2023	2	15	tried	unknown	6-10 times	165
15	38	M	OTP	3/7/2023	2	14	yes	24	20–30 pills	unk
16	52	M	OTP	3/12/2023	intermittent	3	once	12	30–40 pills	0
17	34	F	OTP	3/30/2023	0.1	1	unknown	5	1.5 g	160
18	36	F	OTP	4/26/2023	PW	2	No	0.25	small amount	125
19	27	M	OTP	5/9/2023	0	0.5	no	6	small amount	30
20	37	M	OTP	5/16/2023	intermittent	11	yes	24	1–3 g	65
21	62	M	OBOT	5/22/2023	5	30	yes	12	unkown	20
22	49	M	OTP	5/30/2023	PW	17	yes	12	25–30 pills	0
23	52	M	OTP	6/7/2023	0	36	yes	heroin	1/4 g	130
24	42	F	patient	6/10/2023	intermittent	11	no	24	50–60 pills	0
25	30	F	outreach	6/10/2023	2	16	yes	6	10 pills	0
26	43	F	outreach	6/10/2023	PW	23	yes	24	$20	110
27	40	M	outreach	6/10/2023	0.25	24	no	24	2 times	0
28	54	M	OTP	6/14/2023	intermittent	42	yes	heroin	3 g	0
29	29	M	OTP	6/29/2023	0.04	15	yes	methadone	n/a	90
30	45	F	patient	7/11/2023	1	25	yes	24	1 g	0
31	43	F	OTP	7/11/2023	intermittent	7	yes	1	0.5-1 points	130
32	47	F	patient	7/18/2023	0.1	7	no	24	4 pills	0
33	42	M	outreach	7/20/2023	intermittent	unknown	no	unknown	1.5 g	0
34	55	F	OTP	7/25/2023	intermittent	12	no	unknown	unknown	75
35	40	M	outreach	7/25/2023	0.1	14	unknown	24	unknown	0
36	27	M	OTP	7/28/2023	intermittent	3	yes	12	3.5–7 g	0
37	30	F	OTP	7/28/2023	intermittent	10	no	36	1 g	100

OBOT = Office Based Opioid Treatment; OTP = federally certified Opioid Treatment Program; PW = precipitated withdrawal interfered with previous initiation

*The majority of patients reported diagnoses of depression, anxiety and/or PTSD, and many reported chronic pain. Most were not currently on prescribed medication treatment for these conditions

**Table 2:**
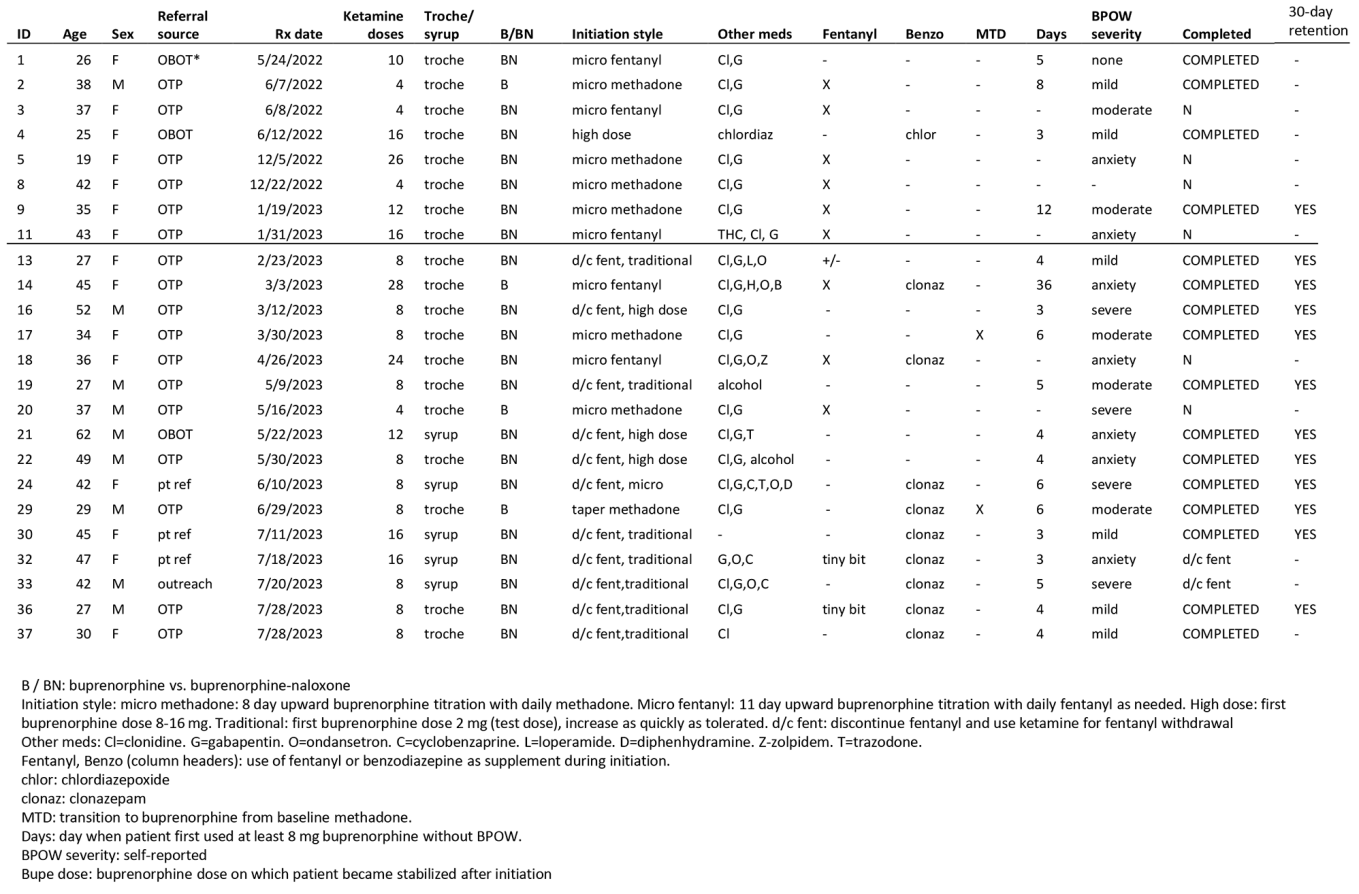
Key treatment elements and outcomes

**Table 3:** Summary of results

	Earlier Patients	Later Patients	Total Prescribed Ketamine	% of Total Prescribed Ketamine	% of Total Who Tried Ketamine
Prescribed ketamine	12	25	37	100%	--
No outcome information	4	9	13	35%	--
Tried ketamine	8	16	24	65%	100%
Did not complete initiation	4	4	8	22%	33%
Completed initiation	4	12	16	43%	67%
30-day retention	1	11	12	32%	50%
XR-BUP	1	4	5	14%	21%

We prescribed ketamine to 37 patients between 5/24/2022 and 7/28/2023. Among these patients, 24 (65%) confirmed trying ketamine and 16 (43%) completed buprenorphine initiation. Among the 24 patients who confirmed trying ketamine, 16 (67%) completed buprenorphine initiation, 12 (50%) achieved 30-day retention, and 5 (21%) initiated XR-BUP. Of all 37 patients, 8 (22%) tried ketamine but did not complete initiation, and 13 (35%) had no outcome information available.

Among the 24 patients who confirmed trying ketamine, buprenorphine initiation was completed by 4 (50%) of the 8-patient “Earlier” group, and 12 (75%) of the 16-patient “Later” group. Among the 12 patients in the “Later” group who completed initiation, 11 (92%) achieved 30-day retention.

Only 1 (20%) of the 5 patients referred by outreach workers reported trying ketamine, while 16 (64%) of the 25 patients referred from the OTP did.

Eleven patients delayed starting buprenorphine up to 72 h after discontinuing fentanyl, using ketamine to control withdrawal symptoms starting day 1; all reported little to no use of fentanyl during that time. Most of these patients reported that ketamine relieved or abolished all fentanyl withdrawal symptoms except anxiety, which was often intense and for which they reported much greater relief from clonazepam than from other supportive medications. Most patients who initiated buprenorphine within 24 h after last fentanyl use reported abrupt onset of mild to severe discomfort interpreted as BPOW. Many patients reported that ketamine reduced severity of symptoms when used as premedication for following buprenorphine doses; some reported that ketamine reduced or abolished symptoms that started after a buprenorphine dose.

The only side effect reported from the ketamine was a perceptual change for about 45 min in two patients who had been instructed to take 48 mg within one hour in an attempt to control BPOW.

We identified a four-day protocol (Table 4) that allowed four patients (#30, #32, #36 and #37) to complete buprenorphine initiation with only mild withdrawal symptoms.

**Table 4:** Procedure for a 4-day initiation with minimal withdrawal (patients #30, #32, #36 and #37)

Day	Premedication	Ketamine + Buprenorphine	Optional
1 and 2	clonazepam 1 mg	Ketamine 16 mg as needed to treat fentanyl withdrawal (1-3 doses per day)	adjunctive medications
3	clonazepam 1 mg	(ketamine 8-16 mg then buprenorphine 2 mg) every 3 h x 4	adjunctive medications
4	clonazepam 1 mg	buprenorphine 4 mg every 3 h x 4	ketamine 8-16 mg premedication, adjunctive medications
5+		buprenorphine 8 mg, repeat as needed up to 32 mg	

## Discussion

Ketamine at a sub-dissociative level with repeated doses self-administered sublingually at home over several days facilitated buprenorphine initiation in the outpatient setting. We describe ketamine treatment in a group of patients who reported previous incomplete buprenorphine initiation attempts, or who were already experiencing intolerable opioid withdrawal symptoms. During an iterative refinement process with prescription of ketamine to 37 patients, we identified key elements of a treatment strategy (Table 4) that allowed four of the last patients to report minimal withdrawal symptoms while completing buprenorphine initiation over four days. The “Later” patients in this series had a higher completion rate compared to the “Earlier” patients, reflecting improvement in the treatment strategy.

Our observations of patient experiences may be useful to others exploring the application of ketamine to facilitate buprenorphine initiation. Most medical providers will have little to no experience with ketamine as a treatment for any indication. Those with ketamine experience will likely be surprised at its potency at such a low dose. One previous report of ketamine for treating opioid withdrawal showed effectiveness in a dose range similar to ours [24]. Three previous reports [23, 25, 26] used a higher dose range at which dissociative symptoms would be expected. Advantages of that higher dose range are unclear.

The protocol we describe is one of many potential strategies. In addition to dissociative-range ketamine infusions in the emergency department [25] and inpatient setting [26], a recent case series of 30 patients at a 24-hour crisis center [29] demonstrated that 10 mg of ketamine delivered by intramuscular injection as premedication 30 min before an 8 mg buprenorphine initiation dose resulted in a drop in average COWS score from 13.7 to 4.0 within one hour. Similarly, sub-dissociative ketamine premedication might reduce severity of BPOW during (1) extended-release buprenorphine initiation [8] and (2) buprenorphine rescue after naloxone self-administration [9].

### Treatment strategy evolution

Based on observations of patients’ experiences and outcomes, we refined the treatment strategy in a stepwise manner to reduce the risk and severity of withdrawal symptoms among successive patients. The changes we made, presented here in chronological order, may have contributed to the more tolerable course and higher completion rate among the “Later” patients:

1. Pretreat prophylactically. Initially, we instructed patients to use ketamine only to treat BPOW symptoms. Patients #2 and #4 discovered that BPOW did not occur or was mild when they took ketamine before a buprenorphine dose. Beginning December 2022 with patient #5, we began instructing patients to premedicate with ketamine before each buprenorphine dose.
2. Avoid a prolonged initiation. Initially, most patients used WCDC’s standard low-dose buprenorphine initiation schedule that titrated up from 0.25 mg/day to 12–16 mg/day over 10 days (or 8 days for transition from methadone to buprenorphine). During these extended initiations, patients’ motivation flagged, monitoring was time-consuming, and ongoing fentanyl use incurred ongoing overdose risk.
  In February 2023, we began to explore strategies to shorten the initiation duration. Patient #13 started buprenorphine 2 mg 4 times daily on day 1, each dose preceded by 8 mg ketamine, and reported only mild withdrawal symptoms. Unfortunately, patient #16, who started with buprenorphine 8 mg on day 1 preceded by ketamine 16 mg, reported severe abrupt-onset withdrawal. Withdrawal symptoms did not respond to treatment with additional ketamine doses totaling 32 mg or an additional buprenorphine 16 mg or adjunctive medications. Patient #21, starting 12 h after last fentanyl use, tolerated buprenorphine 2 mg preceded by ketamine 16 mg. However, an hour later an additional 2 mg produced severe anxiety and vomiting, poorly controlled for several hours despite an additional ketamine 32 mg and buprenorphine 16 mg, plus adjunctive medications. Both patients #16 and #21 eventually recovered and completed initiation.
3. Discontinue fentanyl and use ketamine for fentanyl withdrawal. Initially, most patients continued fentanyl use throughout the initiation period to manage both spontaneous withdrawal and BPOW. Challenges included: (1) BPOW or a state of generalized discomfort (sometimes described as feeling “icky”) continued as long as fentanyl was used, and (2) ongoing fentanyl use obscured initiation completion. Starting in May 2023, we advised patients to designate a fentanyl quit date and time, then abstain from fentanyl for at least 48 h and use ketamine to treat mild to moderate withdrawal symptoms. Ketamine 16 mg markedly reduced or abolished fentanyl withdrawal symptoms for most patients during that time; some patients extended that period to 72 h.
4. Use benzodiazepines to prevent panic attacks. Some earlier patients experienced nonspecific intense distress within one hour after buprenorphine despite treatment with ketamine. These abrupt-onset episodes were often interpreted by the patient as BPOW–though lacking somatic features such as muscle aches or vomiting–and led to treatment discontinuation. Patient #14 discontinued twice for this reason. Ultimately, the patient recognized these episodes as panic attack equivalents. On the third attempt, LG prescribed clonazepam 1 mg orally once daily in the morning for 5 days, and the patient completed initiation without these distressing symptoms. Beginning in June 2023 with patient #24, LG prescribed clonazepam to all patients.
5. Delay introduction of buprenorphine. Based on the undesirable experiences of patients #16 and #21 with early introduction of buprenorphine, and nonspecific discomfort during the first 48–72 h even in patients who denied abrupt-onset symptoms (including #13 above), we attempted the opposite strategy. We instructed patients to delay buprenorphine start until at least 48 h after last fentanyl use, to use ketamine to treat fentanyl withdrawal, and to use clonazepam to prevent panic attacks. That was the final strategy change that led to satisfactory results for four patients using the protocol described in Table 4.

### Additional observations

1. Ketamine dose.
  - *Symptom relief*: Ketamine 16 mg relieved withdrawal symptoms (both spontaneous and BPOW) within 30 min for most patients, with relief lasting hours. Patient #1 started ketamine after 17 h of extreme restlessness, pain in abdomen, back and joints, a “creepy-crawly” sensation, and a feeling of heavy weight. These symptoms completely resolved 5–10 min after ketamine 4 mg.
  - *Cognitive effects*: LG instructed patients #16 and #21 to use ketamine 48 mg total over an hour in an attempt to address BPOW; both were in the company of supportive family members. Patient #16 was frightened by distorted visual effects compounding inadequately controlled BPOW and/or panic. Patient #21 described feeling comfortably dazed for about 45 min.
2. Effectiveness for transition from methadone. We initially aimed to treat only fentanyl-using patients. However, we offered ketamine to Patients #17 and #29 after they reported BPOW during transition to buprenorphine from methadone. The addition of ketamine 16 mg premedication before each buprenorphine dose allowed them to avoid further BPOW. Ketamine’s benefit in treating withdrawal among patients dependent on either fentanyl or methadone is consistent with reports in which ketamine treated withdrawal in patients dependent on unspecified short-acting opioids [23], methadone, hydromorphone or heroin [24].
3. Lack of respiratory depression from subsequent opioid use. Patients #11, #13, #14, and #18 returned for one or more initiation attempts after an incomplete earlier attempt. No overdose occurred after return to fentanyl use despite a theoretical risk of ketamine-induced reduction of opioid tolerance, and each endorsed that use of the previous amount of fentanyl did not produce slowed breathing or excessive sedation.
4. Initiation outcome was associated with referral source. Of five patients referred by outreach workers, outcome information was only available for one; that person successfully discontinued fentanyl but chose not to complete transition to buprenorphine. Discontinuation of fentanyl without initiating buprenorphine may increase risk of overdose death due to loss of opioid tolerance [30]. While our sample size is small, these findings suggest that prescribers should carefully consider whether to offer ketamine treatment to individuals who do not independently present to an OBOT or OTP for treatment.
5. Benefits of daily patient contact. Frequent communication with patients allowed team members to develop therapeutic relationships with them. Some patients expressed appreciation that the support helped them weather the unpredictability of the treatment course. It also allowed for valuable feedback for rapid evolution of the treatment model.

### Potential adverse effects

Adverse reactions to ketamine at the subanesthetic doses commonly used in depression treatment (0.1–0.5 mg/kg) are dose-dependent with regard to both likelihood of occurrence and severity [13]. They can include transient dissociative symptoms, cognitive impairment, and psychotomimetic symptoms. The most common effects are drowsiness, dizziness, poor coordination, blurred vision, and feelings of strangeness or unreality; of greatest concern are elevations of heart rate and blood pressure [13]. Rapid ketamine infusions at an anesthetic dose (1–2 mg/kg) or above could affect any major organ system [10].

Cognitive impairments or anesthesia in a hazardous situation such as near a body of water or on the street alone at night would pose a life-threatening risk. Individuals with impaired decision-making ability such as an uncontrolled bipolar or psychotic disorder might use more than directed and put themselves at risk. However, the dose dependence of even mild adverse effects at the sub-anesthetic dissociative dose range [13] suggests that at the much lower dose range used in this series, the risk of adverse effects from ketamine is lower than that from uncontrolled BPOW or continued illicit opioid use.

Ketamine’s use as a recreational drug suggests diversion risk, and its Schedule III status indicates addiction risk. These concerns should be addressed by carefully assessing patients’ recovery motivation and by limiting the ketamine quantity prescribed. Most of these patients received eight 16-mg doses (bioequivalent to 0.32 mg/kg total), with consequent risk of temporary incapacitation if misused (i.e. if used all at once instead of as directed). A smaller number of doses may be suitable for some patients, particularly in settings with good pharmacy access.

Two patients in this series reported cognitive changes after 48 mg (bioequivalent to ~ 0.17 mg/kg) within one hour that would have put them at risk in a hazardous situation, and one of them found the experience frightening. The authors, based on LG’s experience with hundreds of patients, recommend that dosing for this application in the outpatient setting be limited to 16 mg three times daily, each dose separated by at least three hours, to avoid such experiences.

Of theoretical concern is potential synergy with opioids such that ketamine, like benzodiazepines, might exacerbate opioid-induced respiratory depression and increase risk of overdose death. This concern arises because in rodents [19], ketamine reduces tolerance to the analgesic effect of opioids; it is unclear whether tolerance reduction extends to the respiratory depressive effect of opioids. The limited available evidence suggests it does not. A Cochrane review [31] of 130 randomized controlled trials of ketamine for acute postoperative pain with 8341 participants demonstrated a significant reduction in opioid requirements without an increase in adverse events. No overdose occurred in our small case series, and Patients #11, #13, #14 and #18 denied oversedation with fentanyl use following exposure to ketamine.

A reduction in opioid dependence poses a risk of reduced patient motivation for completion of buprenorphine initiation. Individuals who successfully allay withdrawal symptoms with ketamine for several days may not see a need for ongoing buprenorphine treatment, as reported by Patients #32 and #33, leaving them with lower opioid tolerance and therefore at high risk of overdose if they return to use. On the other hand, the high rate of acceptance of XR-BUP and high 30-day retention may reflect ketamine’s rapid antidepressant effect [13] that perhaps increases patient motivation to avoid illicit drug use.

As discussed in Methods, all of these risks must be weighed against the daily risk of fentanyl overdose death, and might be considered on a case-by-case basis.

## Limitations

Limitations of this report are its subjective nature (absence of objective measurements of patient response or substance use), small sample size, patient heterogeneity, variation in patient instructions and ketamine sources, incomplete information available from patients, and primarily one ketamine prescriber. The potential for misuse or diversion was not evaluated. Selection bias may have contributed to the high completion rate. Generalizability is limited: the protocol we describe may not be suitable in some settings with different constraints. This protocol should not be interpreted as reliably preventing withdrawal symptoms during buprenorphine initiation for all patients; a longer delay before starting buprenorphine, which would require more ketamine doses, may be beneficial for some patients, in part due to prolonged and individual renal clearance of fentanyl [4].

## Conclusion

Ketamine at a sub-dissociative dose allowed completion of buprenorphine initiation in the outpatient setting in the majority of patients who reported trying it. It frequently reduced spontaneous withdrawal symptoms from fentanyl and methadone, and sometimes reduced BPOW when used prophylactically or as treatment, all at a dose that avoided alteration of mental status.

Widespread off-label use of this inexpensive medication to assist buprenorphine initiation could lower the barrier and increase uptake of a life-saving treatment in a high-risk patient population. Further research is warranted to confirm these results and develop reliable protocols for a range of treatment settings.

## Data Availability

All data generated or analyzed during this study are included in this published article.
